# New fossil assemblages from the Early Ordovician Fezouata Biota

**DOI:** 10.1038/s41598-022-25000-z

**Published:** 2022-12-13

**Authors:** Farid Saleh, Romain Vaucher, Muriel Vidal, Khadija El Hariri, Lukáš Laibl, Allison C. Daley, Juan Carlos Gutiérrez-Marco, Yves Candela, David A. T. Harper, Javier Ortega-Hernández, Xiaoya Ma, Ariba Rida, Daniel Vizcaïno, Bertrand Lefebvre

**Affiliations:** 1 Institute of Earth Sciences (ISTE), University of Lausanne, Geopolis, 1015 Lausanne, Switzerland; 2 Yunnan Key Laboratory for Palaeobiology, Institute of Palaeontology, Yunnan University, Kunming, China; 3 MEC International Joint Laboratory for Palaeobiology and Palaeoenvironment, Institute of Palaeontology, Yunnan University, Kunming, China; 4 Univ Brest, CNRS, Ifremer, Geo-Ocean, UMR 6538, Place Nicolas Copernic, F-29280 Plouzané, France; 5 Laboratoire de Géoressources, Géoenvironnement Et Génie Civil ‘L3G’, Faculté Des Sciences Et Techniques, Université Cadi-Ayyad, BP 549, 40000 Marrakesh, Morocco; 6 Czech Academy of Sciences, Institute of Geology, Rozvojová 269, 165 00 Prague 6, Czech Republic; 7 Instituto de Geociencias (CSIC, UCM), Departamento GEODESPAL, Facultad de Ciencias Geológicas, Spanish Research Council, José Antonio Novais 12, 28040 Madrid, Spain; 8 Department of Natural Sciences, National Museums Scotland, Edinburgh, EH1 1JF UK; 9 Palaeoecosystems Group, Department of Earth Sciences, Durham University, Durham, DH1 3LE UK; 10 Museum of Comparative Zoology and Department of Organismic and Evolutionary Biology, Harvard University, Cambridge, MA 02138 USA; 11 Centre for Ecology and Conservation, University of Exeter, Penryn, UK; 12 Université Cadi Ayyad, École Normale Supérieure, Marrakech, Morocco; 13 Independent, 7 rue Chardin, Maquens, 11090 Carcassonne, France; 14 Université Claude Bernard Lyon 1, École Normale Supérieure de Lyon, CNRS, UMR5276, LGL-TPE, Université de Lyon, Villeurbanne, France

**Keywords:** Palaeontology, Sedimentology

## Abstract

The Fezouata Biota (Morocco) is a unique Early Ordovician fossil assemblage. The discovery of this biota revolutionized our understanding of Earth’s early animal diversifications—the Cambrian Explosion and the Ordovician Radiation—by suggesting an evolutionary continuum between both events. Herein, we describe Taichoute, a new fossil locality from the Fezouata Shale. This locality extends the temporal distribution of fossil preservation from this formation into the upper Floian, while also expanding the range of depositional environments to more distal parts of the shelf. In Taichoute, most animals were transported by density flows, unlike the *in-situ* preservation of animals recovered in previously investigated Fezouata sites. Taichoute is dominated by three-dimensionally preserved, and heavily sclerotized fragments of large euarthropods—possibly representing nektobenthic/nektic bivalved taxa and/or hurdiid radiodonts. Resolving whether this dominance reflects a legitimate aspect of the original ecosystem or a preservational bias requires an in-depth assessment of the environmental conditions at this site. Nevertheless, Taichoute provides novel preservational and palaeontological insights during a key evolutionary transition in the history of life on Earth.

## Introduction

**Figure 1:**
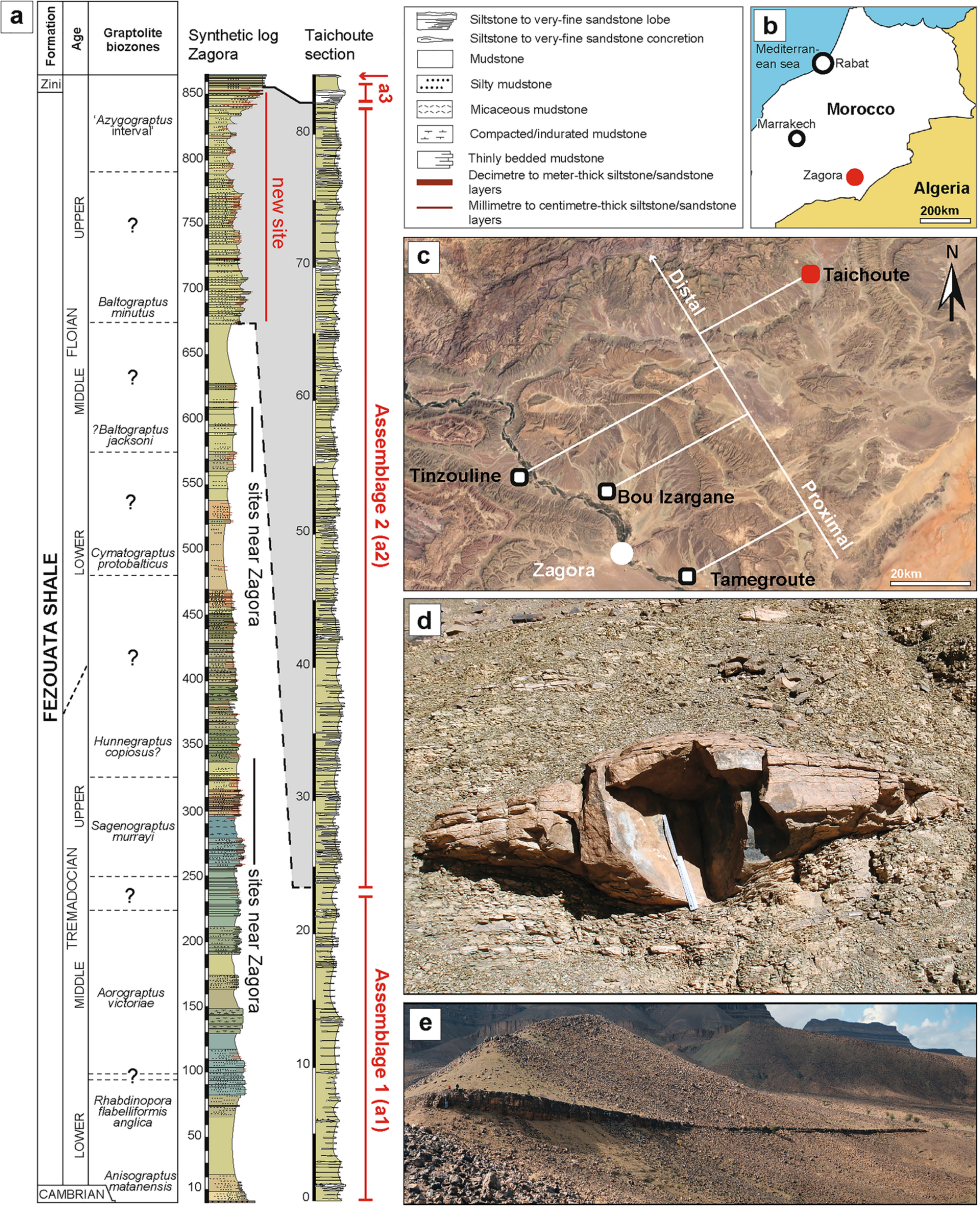
(**a**) Stratigraphic column for the Lower Ordovician Fezouata Shale and Zini formations including the Taichoute locality divided into three fossil assemblages. The red arrow in (**a**) indicates an accumulation level of brachiopods and bryozoans at the top of the Taichoute section. (**b**) Zoom on the Zagora Region where the Fezouata Shale was discovered. (**c**) Taichoute is the most distal site in the depositional environment of the Fezouata Shale. (**d**) Concretion from the Zagora region. (**e**) A large lobe from the Fezouata Shale in the upper part of the succession (a3) and interpreted as a density-flow deposit.

The Early Ordovician Fezouata Biota of Morocco is an exceptionally-preserved fossil assemblage that contains a combination of non-mineralized extinct organisms that have become synonymous with the Cambrian Explosion, such as radiodonts, lobopodians, nektaspidids, and marrellomorphs, alongside more derived forms that are representative of typical Palaeozoic faunas, such as xiphosurans and machaeridians^1,2^. To date, most palaeobiological research^1–25^ has focused on Fezouata fossils that occur in either upper Tremadocian or middle Floian deposits in the Zagora region (Fig. 1a,b)^26^, covering to some extent, the proximal–distal axis of an ancient marine environment (Fig. 1c)^27,28^. This environment was dominated by wave/storm processes^29,30^. The exceptionally well-preserved fossils in these deposits were buried in situ by storm-induced deposits close to the storm wave base (SWB)^29–31^. Lightly biomineralized or sclerotized animals in the Fezouata Biota are most commonly preserved in shales as weathered carbonaceous material (compressed to different degrees) and authigenic minerals^32,33^. Preservation within concretions has also been reported from this formation (Fig. 1d), but this mode of fossilization is more restricted compared to shale-hosted macrofossils^34^. Most Fezouata Shale fossil collections are based on discoveries around the Zagora region in Morocco (e.g., Tamegroute, Bou Izargane, and Tinzouline) (Fig. 1b)^32^; with little effort spent prospecting fossiliferous localities outside this area. Here, we report a new and continuous fossiliferous section from the Fezouata Shale discovered in Taichoute, 80 km away from previously investigated localities (Fig. 1a,c). We discuss the palaeontology, sedimentology, and possible preservational biases operating at this site. The Taichoute locality expands the range of depositional environments yielding non-biomineralized fossils, reveals new modes of preservation for the Fezouata Shale, and expands the occurrence of fossil-bearing strata within this formation.

## Results and discussion

### Faunal content

**Figure 2:**
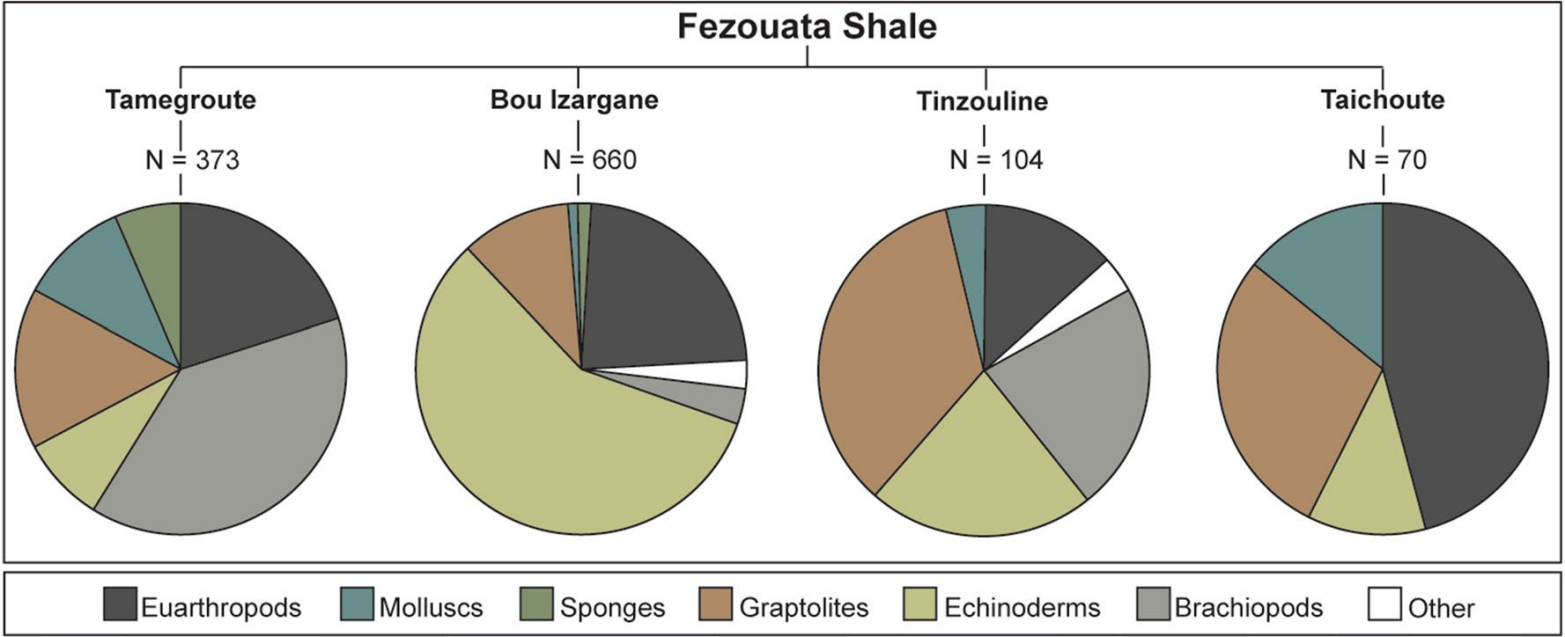
Faunal composition and specimen abundances for each animal group at different Fezouata Shale localities based on the Marrakech Collections of the Cadi Ayyad University (Morocco). Note that the pie chart for Taichoute excludes the accumulation level of brachiopods and bryozoans (above a3, at the top of the succession), as it is impossible to count fossil specimens on this bed. This figure does not account for all specimens recovered/observed at outcrops. For information on how this data was collected and plotted, kindly refer to the Material and Methods section.

Euarthropods, brachiopods, echinoderms, and graptolites constitute together most of the preserved biodiversity in the Fezouata Biota^32^, and this pattern is replicated in the newly described Taichoute locality (Fig. 2). Some animal groups such as sponges, found in some other localities (e.g., Tamegroute and Bou Izargane), remain, so far, absent from Taichoute (Fig. 2).

**Figure 3:**
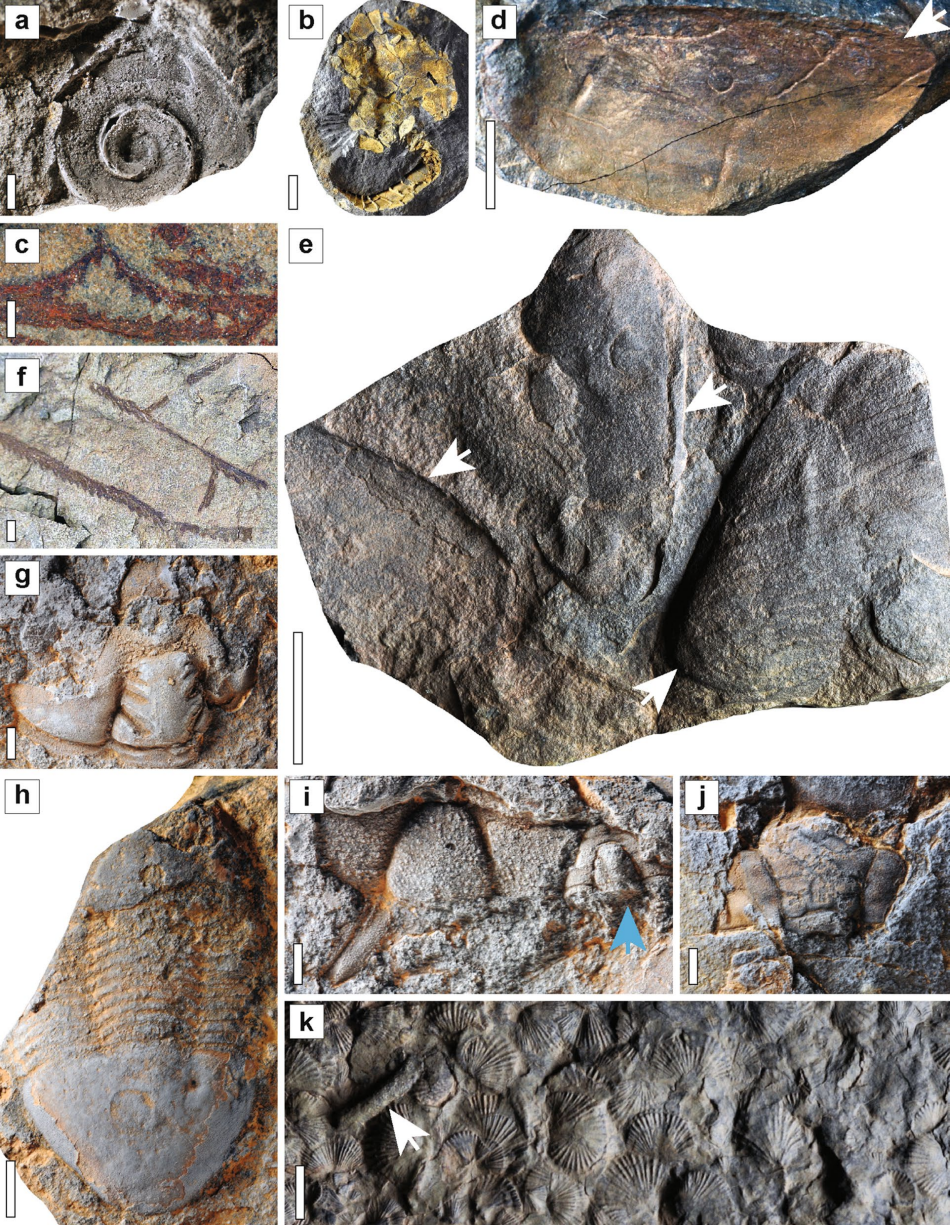
Fossils from the Taichoute locality (a1: A–C, a2: D–F, a3: G–J). (**a**) The gastropod *Lesueurilla prima*. (**b**) The solutan echinoderm *Plasiacystis mobilis*. (**c**) The graptolite *Baltograptus* gr. *deflexus*. (**d**–**e**) Giant euarthropod carapaces indicated with white arrows. (**d**) Carapace likely belonging to previously documented bivalved arthropods from the Fezouata Biota^59^. (**e**) Two incomplete but tapering carapaces (left and center) adjacent to a structure that bears possible resemblance to a block of radiodont setal blades (right), consisting of a series of parallel elongated blades a few millimetres wide separated by slight changes in sediment level and/or by intervening sediment, with an overall tapering outline, similar to the setal blade blocks of hurdiids such as *Hurdia* from the Burgess Shale^60^. (**f**) The multiramous graptolite *Holograptus* sp. (**g**) The calymenid trilobite *Colpocoryphe* cf. *thorali*. (**h**) The illaenid trilobite *Ectillaenus*? sp. (**i**) The calymenid trilobite *Neseuretus* cf. *attenuatus* (blue arrow) and Lichidae gen. indet*.* (to the left of the blue arrow). (**j**) A dalmanitid trilobite, subfamily Zeliszkellinae*.* (**k**) Accumulations of specimens of a new genus of orthidine brachiopod and bryozoans (white arrow) on top of a3. Scale bars = 5 mm in a, f, g, h, i, j; 10 mm in b, c and k; 25 mm in d; 50 mm in e. By order from a to j: AA.TAI2.OI.9; ML20-259,357; AA.TAI3.OI.2; AA.TAI6.OI.1; AA.TAI6.OI.2; AA.TAI6.OI.6; AA.TAI13.OI.1; AA.TAI13.OI.2; AA.TAI13.OI.3; AA.TAI13.OI.4; AA.TAI14.OI.1. All specimens are housed in the Marrakech Collections of the Cadi Ayyad University.

Taichoute can be categorized by three distinct assemblages based on their diversity and preservation (a1, a2, and a3; Fig. 1a). The lowermost assemblage (a1) consists of brachiopods, gastropods (Fig. 3a), echinoderms (Fig. 3b), and graptolites (Fig. 3c). A middle Floian age for assemblage a1 can be inferred by the occurrence of *Baltograptus* gr. *deflexus*^8^ (Fig. 3c). Fossils from this interval are mainly found in concretions that typically contain a single specimen (e.g., Fig. 3b). The preservation of minute details, showing various stages of disarticulation and the *in-situ* collapse of echinoderms, with no evidence of abrasion (Fig. 3b), indicate that these animals were not transported, and concretions formed while organisms were decaying on the seafloor below the SWB in a similar way to some previously described concretion-based preservation from the Zagora region^2^. Another type of concretion, preserving unrecognizable bioclasts transported by storm events and formed above the SWB, has also been previously described from the Zagora region^29^, but has not been found at Taichoute.

**Figure 4:**
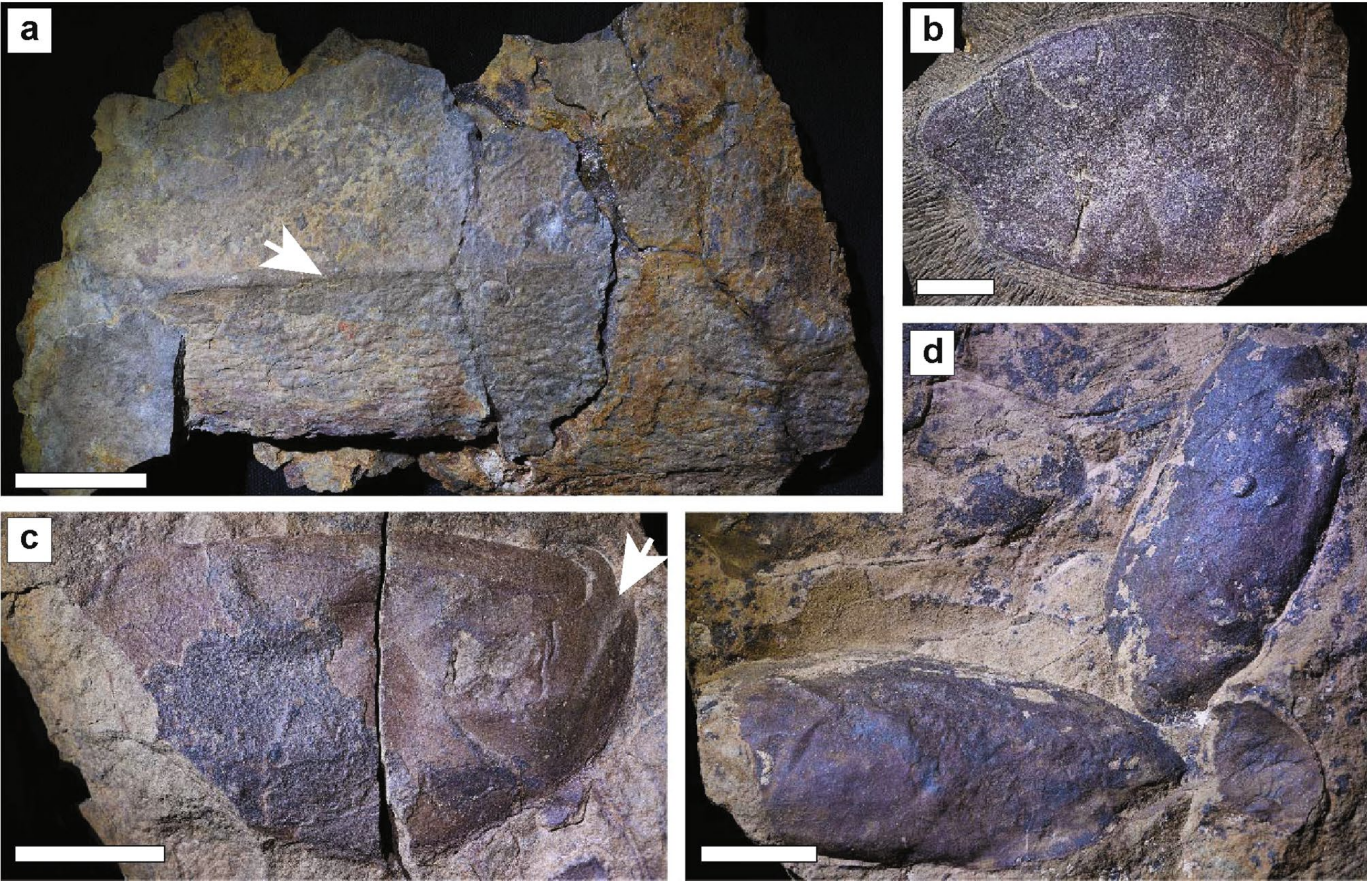
Euarthropod fossils from Taichoute interval a2. (**a**) Large carapace with central carina (white arrow), similar to that seen in *Aegirocassis* dorsal carapace elements^6^. Note the presence of a brachiopod on the right side under the Carina. (**b**) Carapace with an asymmetrically rounded outline with an anterior protrusion similar to that seen in lateral carapace elements of hurdiids^60^. (**c**) Carapace with an asymmetrically rounded outline with one side bearing a structure similar to the upturned projection (white arrow) described for *Aegirocassis* lateral carapace elements^6^. (**d**) Assemblage with at least three carapaces together, some of which are elongated and tapering at one end, with a partial outline possibly resembling that of lateral carapace elements of hurdiids^6^. Scale bars are 4 cm in a, and 3 cm in b, c, d. By order from a to d: MCZ.IP.198899, MCZ.IP.19890, MCZ.IP.198902, and MCZ.IP.198901. All four specimens are housed at the Museum of Comparative Zoology, Harvard University, USA.

The intermediate interval a2 (Fig. 1a) yields concretions with low taxonomic diversity consisting mainly of nautiloid cephalopods, euarthropod fragments (Figs. 3d,e and 4), and graptolites (Fig. 3f), Although there is some variability in their morphology and appearance, the euarthropod carapaces are typically oval-shaped to elongate, and they are preserved with substantial convexity and/or artefacts of previous relief that suggests a heavily sclerotized original constitution (Figs. 3d,e and 4). Coupled with their large size, ranging from ca. 5 to 15 cm in length (Figs. 3d,e and 4), and the presence of thickened marginal rims, we tentatively interpret them as corresponding to bivalved euarthropods and possibly elements of radiodonts (Figs. 3d,e and 4). Whilst preliminary, we argue that radiodont affinities are the most plausible for the fragments considering the known diversity of euarthropods during the Early Ordovician, and the fact that giant hurdiid radiodonts are well known from the Fezouata Shale^5,6^. The observed euarthropod carapaces (Figs. 3d,e and 4) from Taichoute are considerably larger than those described from early- and mid-Cambrian sites [e.g., *Balhuticaris*^35^; *Isoxys*^36,37^; *Tuzoia*^37,38^; *Branchiocaris*^39,40^, and *Tokummia*^41^], and are more comparable in size to stratigraphically younger euarthropods (e.g., phyllocarids) discovered in Silurian and Devonian strata^42,43^.

Bivalved euarthropods and radiodonts are generally interpreted as primarily nektic or nektobenthic components of Palaeozoic ecosystems^1,2,5,6^. The dominance of nektic/nektobenthic euarthropod taxa, nautiloids, and planktonic graptolites in a2 suggests that the seafloor, above which they lived, was not suitable to sustain a diverse and abundant benthic community. However, this environment does not necessarily correspond to Taichoute, mainly because a2 concretions differ from those found in a1 in terms of their quality of fossil preservation. Specimens in a2 are fragmented (Figs. 3d,e and 4). The disarticulated nature of specimens could result from prolonged decay, which may have started in the water column during the bloating and floating stage of initial decomposition, concurrent with processes such as carcass scavenging. Disarticulation may have also resulted from fossil transport, especially given that, unlike a1, a2 concretions are generally not formed of a single specimen, and numerous fragments were often trapped within sediments, that were later consolidated in concretions (e.g., Fig. 3e). Some of these carapaces are covered by meandering traces of varying sizes across their surface, and rare brachiopod epibionts are found on some carapaces (Figs. 3d and 4a) indicating that they could have acted as nutrient sources for the community and/or as a stable substrate for brachiopods to attach to, following their transport to Taichoute, and prior to their consolidation in concretions in a2^44^.

The upper interval a3 (Fig. 1a) consists of a large sedimentary lobe yielding fragmented bryozoans and trilobites (Fig. 3g–j). These large sedimentary bodies in the Fezouata Shale (Fig. 1e) are considered as the stratigraphically distal equivalent to the overlying proximal Zini Formation (Fig. 1a). Due to the degree of fossil disarticulation and fragmentation within this lobe, it is certain that carcasses were transported prior to their preservation. The uppermost interval, above this lobe shows an accumulation level preserving rhynchonelliform brachiopods and bryozoans in minute detail (on planar bedding surfaces, and not within concretions) with very little evidence of physical abrasion (Fig. 3k).

### Sedimentary environment

**Figure 5:**
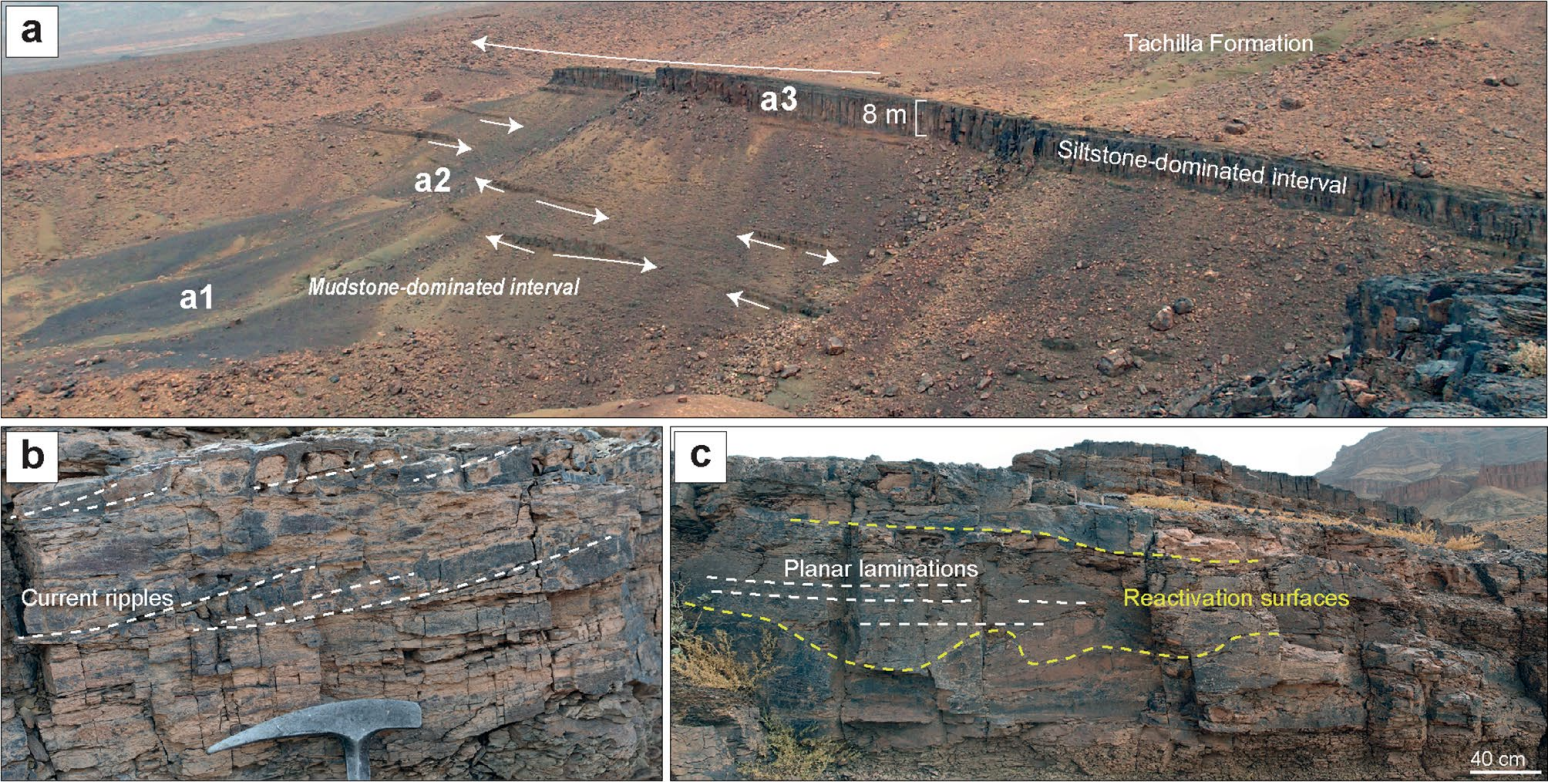
Sedimentary context of Taichoute. (**a**) Overview of the upper part of the Fezouata Shale displaying a transition between a1, a2, and a3. The large siltstone-lobes of a3 transition upward into the overlying Middle Ordovician Tachilla Formation. The white arrows show the lateral discontinuity, characteristic of the siltstone lobes. (**b**) The asymmetrical cross-laminations form current ripples displayed in the siltstone lobes. (**c**) Reactivation surfaces occur frequently in the siltstone lobes, attesting to the turbulence of repeated density flows. These deposits also display planar laminations.

**Figure 6:**
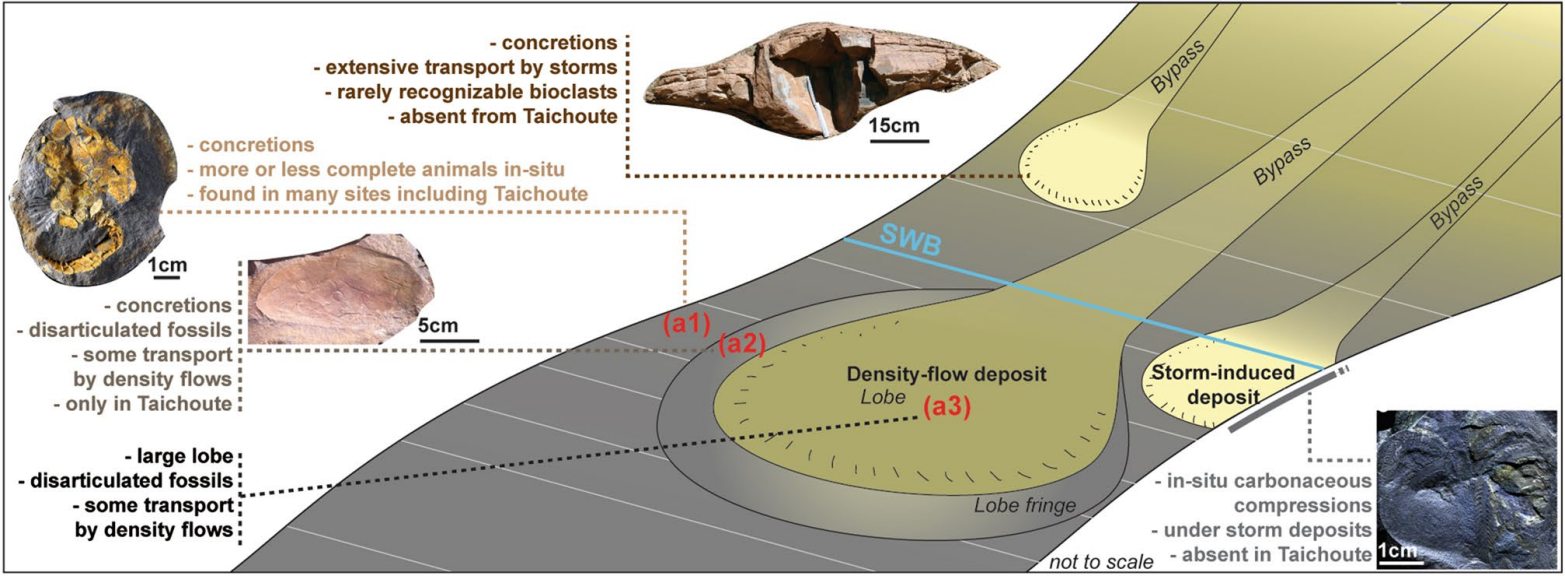
The different modes of preservation within the Fezouata Shale, their distribution, and the sedimentary processes at play for their formation. SWB: Storm Wave Base.

The Fezouata Shale sequence shallows upwards, but this is mainly evident in the Zagora area, where it ends with the deposition of the Zini Formation (Fig. 1a), interpreted as nearshore sandstones^30,45^. However, the entire environment represents a proximal-to-distal gradient from the southeast (in Algeria) to the northwest^30,46^. Thus, the shallowing-upwards sequence is not expressed in terms of facies everywhere in the Fezouata Shale. In this formation, the position of large siltstone lobes, similar to the one deposited in a3, reflects an increase in the sediment supply into the basin linked to a lowering of the base level and an increase of erosion in the shallower-water south-eastern area. These large hectometric lobes that are siltstone-dominated (Fig. 5a) with minor very fine sandstone components, and displaying dominantly planar lamination, asymmetrical cross-laminations, and reactivation surfaces (Fig. 5b,c), have been described as storm-induced, density-flow deposits in the most distal setting of the Fezouata Shale^30^. The interpretation of Taichoute as the most distal section is in accordance with its position in the basin and the previously defined proximal–distal axis^30^ (Fig. 1c). This study is the first to document fossil preservation in association with such settings in the Fezouata Shale, regardless of the fidelity or quality of this preservation. Considering the deposition continuity between a1, a2, and a3 (Fig. 5a), it is likely that all three intervals represent lateral variations of deposition, from normal sedimentation on the seafloor (a1), passing through lobe fringe (a2) to lobe centre (a3) within the same shelf setting (Fig. 6).

These sedimentological interpretations complement previous studies made on the Fezouata Shale that highlighted a difference in the mechanism of fossil preservation between this Ordovician site and other Cambrian deposits preserving labile anatomies such as the Chengjiang Biota (China) and the Walcott Quarry in the Burgess Shale (Canada). It was suggested that most animals preserved near the Zagora region were preserved *in-situ*^13,29,31^ with little to no evidence of transport following death, unlike the aforementioned Cambrian sites where sedimentary flows, particularly turbidity currents, were operational, transporting animals from their living environment to facies favourable for their preservation^47–50^. The discovery of Taichoute increases the similarity between the Fezouata Shale and some Cambrian sites by showing that carcasses could be transported within the general context of this formation. However, the Fezouata Shale remains a unique formation with various modes of fossilisation including animal preservation (i) in accumulation levels (above a3 in Taichoute) (Fig. 1a) (ii) as bioclasts in concretions deposited above the SWB (present near Zagora); (iii) in shales around the SWB (dominant near Zagora), and (iv) under the SWB: (a) *in-situ,* in concretions of different sizes (similar to a1 in Taichoute and some sites near Zagora), (b) transported, in concretions (similar to a2 in Taichoute), and (c) transported, in large hectometric lobes (similar to a3 in Taichoute) (Fig. 6). This taphonomic variability is likely driven by the wide spatial and temporal distribution of the Fezouata Shale, around 850 m of sediments (Fig. 1a) over 900 km^2^ in the Draa Valley, spanning ~ 11 myr. This suggests that the Fezouata Shale is an ideal candidate to further investigate a possible continuum between shale and concretion preservation, and untangle the possible environmental conditions responsible for the transition between these two modes which preserve most of exceptionally preserved fossils during the Early Palaeozoic.

### Possible taphonomic biases

Non-biomineralized fossils in Taichoute are restricted to large euarthropod carcasses, consisting of heavily sclerotized material, preserved in substantial three-dimensional relief, to the exclusion of more delicate structures such as body cuticles, cellular sheets in contact with the water column, and internal organs (Fig. 3). As such, it is not possible to exclude a taphonomic filter^51–54^ at play in Taichoute. This filter could result from a prolonged exposure to decay as both biomineralized and heavily sclerotized structures are more resistant to degradation than other tissue-types^51^. Furthermore, it was previously shown that big carcasses provide large quantities of decaying organic material favouring concretion growth^28,34^. This resulted in the in situ preferential preservation of large-bodied remains in concretions in some sites near Zagora^28,34^. In Taichoute, large animals were transported and trapped within sediments, that were later consolidated in a2. It is possible, that the organic material generated from these carcasses following their transport, facilitated siltstone (i.e., interpreted as lobe-fringe deposits; Fig. 6) consolidation around large carcasses. The mineralogy of the surrounding matrix might have also played a role controlling what got preserved in Taichoute. Particular mineralogical phases are associated with horizons with soft-tissue preservation in the Fezouata Shale^55,56^ and in other deposits bearing exceptional preservation around the world^57,58^. These minerals replicate soft tissues, provide resistance to decay, and induce damage to bacterial membranes, facilitating the preservation of labile structures in the fossil record. In this sense, the absence of labile tissues and dominance of heavily sclerotized structures could also point to a lack of these favourable mineralogical phases in Taichoute sediments.

## Conclusion and perspectives

Taichoute is a newly described middle to late Floian fossil site in the Fezouata Shale. This discovery contributes to our understanding of animal preservation in this unique Lower Ordovician formation by showing that some organisms are transported by density flows. Three types of concretion preservation exist in Taichoute, expanding the fossilization modes observed within the Fezouata Shale. The dominant concretion type preserves large euarthropod carcasses, which, based on their morphology and size, might correspond to either bivalved forms and/or possibly radiodonts. Future mineralogical and geochemical investigations on Taichoute have the potential to produce valuable preservational insights allowing us to resolve whether the aforementioned ecological observations represent legitimate aspects of original ecosystems, or are biased by taphonomic filters. Nevertheless, at this stage, and prior to yielding further investigations on Taichoute, this site remains a significant fossil locality providing novel sedimentary, taphonomic, and palaeontological information (Table 1) at a key interval in the history of life on Earth—at the transition between the Cambrian Explosion and the Ordovician Radiation.

**Table 1:** Comparison between the newly discovered Fezouata Shale locality (Taichoute) and conventional sites.

	Traditional Fezouata Shale sites	This study (Taichoute)
Location	Zagora Region, Morocco	Taichoute, 80 km NNE of Zagora, Morocco
Age	Late Tremadocian to middle Floian	Middle to late Floian
Environment	Dominated by tempestites	Dominated by storm-induced density flows
Transport	Limited, mainly in situ	Pronounced in most of the section
Fossil matrix	Shales (relatively rare concretions)	Concretions of different types and sizes
Preservation fidelity	Biomineralized, sclerotized, and cuticularized structures, and internal organs	Biomineralized structures, and heavily sclerotized large euarthropod fragments (new taxa)

## Material and methods

In 2017, a field excavation took place in Taichoute (Lower Ordovician, Morocco), 80 km away from more traditional Fezouata localities (Fig. 1c). The 85 m-thick sedimentary succession at Taichoute was logged at a dm-scale, taking into account, sedimentary structures, grain size, and bed geometries. Over 300 fossil samples were studied locally, but only 70 samples were transported to the Marrakech Collections of the Cadi Ayyad University in Morocco owing to the heavy weight of the concretions and are accounted for in Fig. 2. Kindly note that more than 12,000 specimens were transported from Bou Izargane to the Marrakech Collections, but only 660 specimens were identified and inventoried so far; these are as such considered in Fig. 2. We acknowledge that taxonomic abundances are likely to change following the complete curation of specimens from Bou Izargane and new fossil discoveries at Tamegroute, Tinzouline, and Taichoute, and these abundances are also likely to fluctuate between fossil collections of different institutions (e.g., Cadi Ayyad University, Yale Peabody Museum, University of Lausanne, and Harvard University). For these reasons, abundances are not central to this manuscript. However, it is important to emphasize that abundance discrepancies between collections and fossiliferous localities do not influence the Taichoute conclusions made within this paper (i.e., location, age, environment, preservation). Numbered material in Fig. 3 is deposited at the Cadi Ayyad University in Morocco, while fossils in Fig. 4 are deposited in the Invertebrate Paleontology collections at the Museum of Comparative Zoology, Harvard University (MCZ.IP).

## Data Availability

All data needed to evaluate the conclusions in the paper are present within the *Main Manuscript*. Additional data related to this paper may be requested from the corresponding author (F. Saleh: farid.nassim.saleh@gmail.com).
